# The Anatomy and Surgical Treatment of Abdominal Hernia: With Numerous Plates

**Published:** 1844-05

**Authors:** 


### BIBLIOGRAPHICAL NOTICES.

---

ART. IX.—*The Anatomy and Surgical Treatment of Abdominal Hernia:* with numerous Plates. By SIR ASTLEY COOPER, Bart., F.R.S., Surgeon to the King, and Consulting Surgeon to Guy's Hospital. From the Second London Edition. By C. ASTON KEY, Senior Surgeon of Guy's Hospital and Lecturer on Surgery. Philadelphia: Lea & Blanchard; pp. 427, large 8vo; 1844.

It would be somewhat late to praise a work which for nearly forty years has ranked among the authorities of the profession, and upon which in part rests the fame of its world-renowned author as a consummate surgeon and anatomist. At the time of its first appearance, in 1804,* it must indeed have been a most remarkable production. The anatomy of hernia was ill understood, or rather was not understood at all, the only author who had made any researches on it being Camper, whose *Icones Herniarum* were published in 1801, after his death. Anatomists had described, what is now known as the external abdominal ring, as being closed behind directly by the peritoneum. Even so late as 1785, Richter, whose work, according to Lawrence, is valuable for its clear narration of symptoms, &c., thus describes it. It was reserved for Cooper to place this important matter in its true light; and the anatomy of the parts, as described by him,

---

* The work originally consisted of two parts, published at different times. The first part, under the title of "*Anatomy and Surgical Treatment of Inguinal and Crural Hernia,*" appeared in 1804; the second, entitled "*Anatomy and Surgical Treatment of Crural and Umbilical Hernia,*" was published in 1807. In 1827, the second edition, under the present title, was given to the public by Mr. Key. All these were in folio.

the result of careful and elaborate dissection, is now very generally acknowledged to be correct.

We owe also to Cooper the rule for operating, applicable to every species of hernia, viz: to cut directly upwards in dividing the stricture; a precept which, he tells us, was taught publicly by him as far back as 1792. But we need not enumerate all the improvements introduced by him. We hope every one will provide himself with a copy of the book; and not rest satisfied with the meagre extracts from it to be found in surgical treatises. An attentive perusal of it will give clearer ideas of the subject, than all these together.

The work is essentially practical. The author scarcely quotes the opinion of a single writer, not from a desire to slight or undervalue the labors of any, but because he wished to confine himself to the unequalled stores furnished by the Hospitals of Guy and St. Thomas, and to the observations collected from private practice in the metropolis. Hence he gives no case for the truth of which he cannot vouch. Another reason for this may be found perhaps in the fact that Cooper, like our Physick, was not a man of much learning, or of much reading, but possessed a rare practical sagacity, and a wonderful self-reliance.

The plates of the folio edition are all preserved in this, although of course reduced in size. Great care has been bestowed by the publishers to insure accuracy in this reduction, as well as correctness of the references. The execution of the plates is worthy of all praise.

We may state here, that the same house, Messrs. Lea & Blanchard, have it in contemplation, to republish the other works of Cooper in the same beautiful style. We hope the profession will evince their approbation of this scheme, and their appreciation of the merits of the works, in the only practicable way.

Cooper, as our readers are aware, died about two years ago at an advanced age. One would suppose that such a man could depart in peace, looking back as he might and seeing his name written in characters of light on some of the brightest pages in the records of surgery. C.

